# Exploring the relationships between personality and color preferences

**DOI:** 10.3389/fpsyg.2022.1065372

**Published:** 2022-12-19

**Authors:** Juliet Jue, Jung Hee Ha

**Affiliations:** ^1^Department of Art Therapy, Hanyang Cyber University, Seoul, Republic of Korea; ^2^Graduate School of Counseling Psychology, Hanyang University, Seoul, Republic of Korea

**Keywords:** personality, big five personality, color, color preference, color connotation

## Abstract

**Introduction:**

This study set out to quantitatively examine the relationship between personality and color, focusing on connotations and preference.

**Method:**

A total of 854 Koreans, aged from 20 to 60, participated in the study. They indicated which colors they associated with various personality words, completed the Ten Item Personality Inventory, and ranked their color preferences. We analyzed the data using frequency analysis, correlation analysis, *t*-tests, regression analysis, and cluster analysis.

**Results:**

The analyses revealed that all five personality types have characteristic color associations. Through regression analysis, we found that color preference can significantly predict personality. The comparison among personality groups produced by cluster analysis confirmed that people with strong specific personalities prefer the colors that symbolize their personalities.

**Discussion:**

This study’s findings highlight the relationship between personality and color preference. The limitations and suggestions for future studies are also presented.

## Introduction

Inter-individual differences in color preference have been noted by a number of studies (Hurbert and Ling, 2007; Lee et al., 2009; Al-Rasheed, 2015; Fetterman et al., 2015; Tao et al., 2015). Previous research found how much people prefer different colors according to their culture, gender, and age group (Palmer and Schloss, 2015). For instance, preference for red especially vary by culture (Hurbert and Ling, 2007; Al-Rasheed, 2015). The Chinese tend to prefer red (Zhang et al., 2019), showing a much higher preference for it than the British (Hurbert and Ling, 2007). In addition, a study comparing the color preferences of the British and Arabs showed that the British prefer blue and Arabs prefer red (Al-Rasheed, 2015). Meanwhile, a study of gender differences in color preference found that females have a strong preference for reddish colors, while males prefer green–blue (Hurbert and Ling, 2007).

Recently, color researchers have devoted more attention to the relationship between color and personality (Kim, 2005; Kim and Park, 2008; Cha et al., 2009; Je et al., 2011; Kim and Kim, 2013; Fetterman et al., 2015; Tao et al., 2015; Cha and Jung, 2018; Lee and Lee, 2021). Most of the studies have been successful in finding associations between personality and color preference, which can be explained in terms of color-evoked emotions. As colors have three dimensions (active–passive, light–heavy, and cool–warm), they could evoke certain emotions accordingly (Ou et al., 2004). However, it is still challenging, as there have been exceptions (Kim and Park, 2008; Kim and Kim, 2013; Cha and Jung, 2018). For example, since red is a vibrant, energetic, warm, and vigorous color, it has been widely believed that people who prefer it are similarly energetic and enthusiastic (Verner-Bonds, 2000). However, recent empirical studies have shown that thinking-type people who prioritize logic and objectivity prefer red color to blue, while blue is widely perceived as symbolizing logic (Gage, 2000; Kim and Kim, 2013; Jue, 2017; Cha and Jung, 2018). Similarly, Kim and Park (2008), who used the introvert-extrovert personality classification, found that introverts prefer red much more than extroverts. Introverts tend to direct their attention inward, which runs contrary to the widely known characteristics of red–action, power, and dominance (Andrews, 2005; McLeod, 2006). These results raise the need to examine the relationship between color impressions and the personalities.

This study set out to investigate color impressions in the general public and to elucidate the relationship between personality type and color preference. Previous studies of the relationship between personality and color preferences have used personality classifications such as Myers–Briggs Type Indicator (MBTI; Kim, 2006) and Ennergram (Lee and Lee, 2021) to examine personality types, or psychological states including depression and/or anxiety (Cho and Oh, 2016). In this study, we classified personality types based on the Five Factor Model, which is commonly used in personality research (Grice, 2019). Using words that describe the Big Five Personalities to examine color associations enabled us to approach the relationship between color impressions and personality from various angles.

## Materials and methods

### Participants

A total of 854 Korean adults responded to the survey. Participants read research participation brochures online and voluntarily participated in the study. The surveys were conducted online only. The gender ratio of respondents was 474 women (55.5%), 355 men (41.6%), and 25 (2.9%) others. Their average age was 32.0 years (S.D. 7.7 years), ranging from a minimum age of 20 to a maximum age of 60.

### Measures

#### Ten item personality inventory

We used the Ten Item Personality Inventory (TIPI) developed by Gosling et al. (2003). The TIPI is the most brief measure of the Big Five Personality domain. It consists of a total of 10 items, and each item has two adjectives. It is rated on a seven-point Likert scale. Two items per personality type consist of adjectives describing the extremes of said personality; one item shows the core of that personality, and the other shows the opposite trait of that personality. Thus, the opposite items are reverse-scored. The test–retest correlation reported by Gosling et al. was *r* = 0.72, and the convergent correlation was *r* = 0.77.

#### Color stimuli and preference calculation

We used the 10 primary colors described by Zhang et al. (2019) and Kim (2006), including red, orange, yellow, green, light blue, dark blue, purple, white, black, and gray. We chose these colors for the following reasons. First, although colors can be presented in many different ways depending on hue, chroma, or brightness, research regarding color preference has mainly focused on hue manipulation and shown that people prefer high-chroma colors when presented with colors without objects (Ou et al., 2004; Pazda and Thorstenson, 2018). In addition, considering that previous studies investigating color preference have found that varying brightness and saturation does not affect hue preference (Hurbert and Ling, 2007), we did not manipulate brightness or saturation levels. Instead, we presented typical hue category colors with high chroma, rainbow colors. The advantage of using the rainbow color array is that it is the most familiar color composition for lay people (Kim, 2006).

Ten square color pieces and their color names were presented together, and respondents were asked to choose the color that most closely matched their preference. For example, if they wanted to choose reddish purple, they would choose purple. Colors were ranked from 1st to 10th, and we revers-scored the color rankings on a scale of 1–10. In other words, first ranked colors received 10 points, second ranked colors received nine points, etc. Therefore, the higher the score, the stronger the color preference.

### Procedures

This study passed the IRB review of the researcher’s institution. We post flyers online, soliciting participation. When participant candidates clicked the survey link in the flyer, they saw an overview of the study, a list of their rights as participants, and a survey manual. After they provided their consent to participate in the study, the survey started. In this study, we did not collect personal information that could identify the participants, except their ages and genders.

A total of 10 colors were presented as color pieces and words. People ranked their favorites among these colors in order from 1st to 10th. If the exact colors that the participants wanted were not available, they were asked to choose the closest match. Next, the TIPI test was performed. Participants read sentences describing personality types and indicated on a seven-point scale how similar or dissimilar the types described in the sentences were to their personalities.

After the TIPI test, 20 personality adjectives from the TIPI test were presented one by one, and participants were asked to choose the colors that best matched each word. Participants could choose a minimum of one color and a maximum of three colors per word. For example, when “extraverted” was presented, a participant could respond with between one and three colors, such as red, yellow, and orange. We provided participants no information regarding the character of each word.

### Analysis method

We analyzed the data using SPSS 26.0, conducting frequency analysis and calculating the percentages and descriptive statistics for each color preference and participant description. We also performed correlation analysis between personality types and colors. Next, we divided personality types into upper and lower groups (e.g., lower < M–1SD, and M + 1SD < upper) and conducted a *t*-test to examine the differences in color preferences between the two groups.

To obtain a more holistic picture of the interrelationships, we converted the original scale scores to standardized scores and performed a multiple regression analysis. In the regression analysis, color preference was set as the independent variable and the traits were the dependent variables.

Finally, using standardized scores, we performed cluster analysis on trait scores, and then compared them in terms of color preference. Cluster analysis is a multivariate analysis technique in which similar objects are grouped together, and each group formed at this time is called a cluster. There are hierarchical and non-hierarchical methods for cluster extraction. The former is performed first, and then the latter is performed to determine the cluster to which each individual belongs. In the hierarchical cluster analysis, the distance between clusters was calculated using Ward’s method, and in the non-hierarchical cluster analysis, the K-means method was implemented. After determining the optimal number of clusters through a dendrogram, an (ANOVA) and a *pos hoc* comparison were performed to verify the difference between preferred colors according to each cluster.

## Results

### Colors associated with personality words

Table 1 presents the results of our examination of the associations between colors and the personality adjectives. The frequency analysis revealed that participants chose the same or similar colors for two words indicating one personality type, even when the personality words were presented separately. For example, the highest proportion of participants chose red as the color associated with the two words describing extraversion—extraverted and enthusiastic. Likewise, for agreeableness (sympathetic and warm), the highest proportion of participants selected yellow, while for emotional stability and sincerity, they ranked green as color most strongly associated with the personality words.

**Table 1:** Colors associated with personality words.

Personality type	Adjective	First ranking (%)	Second ranking (%)	Third ranking (%)
Extraversion	Extraverted (+)	Red (24.7)	Yellow (19.3)	Orange (12.8)
	Enthusiastic (+)	Red (43.2)	Orange (16.4)	Yellow (11.1)
	Reserved (−)	White (16.2)	Dark blue (15.2)	Black (14.5)
	Quiet (−)	White (22.6)	Gray (15.4)	Green (14.8)
Agreeableness	Sympathetic (+)	Yellow (24.3)	Green (19.4)	Orange (16.6)
	Warm (+)	Yellow (27.2)	Orange (24.6)	Red (12.8)
	Critical (−)	Black (21.3)	Gray (20.0)	Dark blue (13.2)
	Quarrelsome (−)	Red (32.5)	Black (13.6)	Purple (9.5)
Conscientiousness	Dependable (+)	Green (25.0)	Light blue (21.6)	Dark blue (16.2)
	Self-disciplined (+)	Green (17.7)	White (17.5)	Dark blue (15.7)
	Disorganized (−)	Purple (22.7)	Gray (17.5)	Red (13.7)
	Careless (−)	Red (22.0)	Orange (12.1)	Purple (11.7)
Emotional stability	Calm (+)	Green (18.0)	White (16.6)	Dark blue (15.1)
	Emotionally stable (+)	Green (24.6)	Light blue (15.6)	White (13.9)
	Anxious (−)	Purple (21.7)	Red (17.9)	Gray (14.5)
	Easily upset (−)	Red (44.0)	Orange (12.5)	Yellow (8.9)
Openness to new experiences	Open to new exp. (+)	Green (19.2)	Yellow (19.0)	Light blue (18.8)
	Complex (+)	Purple (28.5)	Gray (15.0)	Dark blue (11.1)
	Conventional (−)	Black (30.6)	Gray (18.0)	White (11.8)
	Uncreative (−)	Black (31.0)	Gray (22.0)	White (16.2)

The + symbol shows the core of that personality, and the – symbol shows the opposite trait of that personality.

### Correlation analysis results

We conducted a correlation analysis to examine the relationship between personality types and color preferences (see Table 2). While the analysis showed no significant correlation between extroversion and color preference, the remaining four personality types showed significant correlations with specific colors. First, it revealed a positive correlation between agreeableness and preference for yellow, light blue, and white, and a negative correlation between agreeableness and preference for red. Second, the analysis also showed a positive correlation between conscientiousness and preference for light blue and dark blue, and a negative correlation between conscientiousness and preference for red. Meanwhile, emotional stability was positively correlated with preference for light blue, dark blue, and white, and negatively correlated with red and yellow. Finally, open to new experiences was positively correlated with preference for bright blue and white, and negatively correlated with preference for orange.

**Table 2:** Correlation coefficients and descriptive statistics for measurement variables.

		Extra-version	Agreeableness	Conscientiousness	Emotional stability	Open to new experiences
	M (S.D.)	7.7 (2.2)	9.1 (2.1)	9.4 (2.2)	9.0 (2.2)	9.2 (2.1)
Red	6.5 (3.1)	0.04	−0.10^**^	−0.07^*^	−0.16^***^	−0.05
Orange	5.9(2.8)	−0.03	0.01	−0.06	0.06	−0.08^*^
Yellow	6.5 (2.6)	−0.02	0.08^*^	−0.02	−0.08^*^	−0.04
Green	6.7 (2.4)	0.06	0.03	0.05	−0.04	0.06
Light blue	6.5 (2.4)	0.02	0.12^***^	0.13^***^	0.13^***^	0.07^*^
Dark blue	5.7 (2.5)	−0.01	0.06	0.07^*^	0.11^**^	0.06
Purple	4.9 (2.5)	0.03	0.05	0.00	0.03	0.06
White	5.1 (2.6)	0.00	0.08^*^	0.05	0.09^**^	0.10^**^
Gray	3.8 (2.6)	−0.05	−0.03	0.03	0.05	0.11
Black	4.0 (2.9)	−0.06	−0.05	−0.00	0.06	−0.00

^*^p < 0.05, ^**^p < 0.01, and ^***^p < 0.001.

### Comparison between the two personality type groups

Next, we explored the relationship between the respondents’ personalities and color preferences by comparing the upper and lower personality groups (see Table 3). The group with strong extroversion had a stronger preference for green than the group with low extroversion. Those with high agreeableness preferred yellow, and those with high conscientiousness preferred light blue and dark blue. When the emotional stability was high, the preference for red was lower, and the preference for bright blue was higher. Finally, those with strong openness to new experiences preferred green.

**Table 3:** Comparison between the two personality type groups.

Personality	Color	Low	High	*t*
Extraversion		(*N* = 139)	(*N* = 174)	
	Green	6.4	7.0	−2.1^*^
Agreeableness		(*N* = 87)	(*N* = 146)	
	Yellow	5.8	6.7	−2.6^**^
		(*N* = 166)	(*N* = 175)	
Conscientiousness	Light blue	6.2	7.1	−3.3^***^
	Dark blue	5.8	6.3	−2.0^*^
		(*N* = 92)	(*N* = 133)	
Emotional stability	Red	6.6	5.6	2.5^**^
	Light blue	6.3	7.0	−2.1^*^
Open to new experiences		(*N* = 82)	(*N* = 136)	
	Green	6.3	7.0	−2.0^*^

^*^p < 0.05, ^**^p < 0.01, ^***^p < 0.001.

### Regression analysis results

To verify whether color preference significantly predicts personality traits, we conducted multiple regression analysis with color preference as the independent variable and personality traits as the dependent variables. All data used were standardized scores, and the enter method was used for the analysis. The results of multiple regression analysis are presented in Table 4.

**Table 4:** The effect of color preference on big five personality trait.

Dependent variable	Independent variable	*R*	*R^2^*	*F*	B	SE	*β*	*t*
Extraversion	-	0.13	0.02	1.31				
Agreeableness	Yellow Light blue White	0.22	0.05	4.18^***^	0.11 0.10 0.14	0.04 0.04 0.04	0.11 0.10 0.14	2.75^**^ 2.72^**^ 3.38^**^
Conscientiousness	Light blue	0.16	0.03	2.25^*^	0.11	0.04	0.11	2.91^**^
Emotional stability	Red Light blue	0.21	0.05	3.95^***^	−0.12 0.12	0.04 0.04	−0.12 0.12	−2.98^**^ 3.13^**^
Open to new experiences	Green Purple White	0.18	0.03	2.88^**^	0.08 0.07 0.13	0.04 0.04 0.04	0.08 0.07 0.12	2.07^*^ 2.01^*^ 2.98^**^

In the case of independent variables, only those found to be significant in the regression analysis were presented. ^*^p < 0.05, ^**^p < 0.01, and ^***^p < 0.001.

We found that color preferences significantly predicted all personality traits except extraversion. Agreeableness was significantly predicted by yellow, light blue, and white preferences. It was found that the preference of light blue significantly predicted conscientiousness. Emotional stability was significantly predicted by red and light blue preferences. Openness to new experiences was predicted by green, purple, and white preferences.

### Cluster analysis and ANOVA results

After standardizing the TIPI personality test results, the hierarchical cluster analysis was performed using Ward’s method. As a result of checking the dendrogram, we decided to set the number of clusters to five. Based on the five clusters, we conducted the K-means method, which is widely used among nonhierarchical clustering methods. The results of the final cluster classification are presented in Table 5. The five cluster characteristics were named as follows: anti-conscientiousness, emotional stability, extraversion, openness to new experiences, and agreeableness.

**Table 5:** Cluster analysis results.

Personality traits	Cluster 1 (*N* = 333)	Cluster 2 (*N* = 85)	Cluster 3 (*N* = 108)	Cluster 4 (*N* = 119)	Cluster 5 (*N* = 209)
**Cluster’s name**	**Anti-conscientiousness**	**Emotional stability**	**Extraversion**	**Open to new experiences**	**Agreeableness**
Extraversion	−0.01	−0.65	1.14	0.91	−0.85
Agreeableness	−0.55	−0.77	−0.11	0.61	0.93
Conscientiousness	−0.74	0.77	−0.58	0.91	0.68
Emotional stability	−0.58	0.93	−0.74	0.83	0.49
Open to new experiences	−0.68	−0.38	0.45	1.29	0.27

Next, ANOVA was performed to determine if there was a difference in color preference for these five clusters. The results are presented in Table 6. The differences between groups were significant for the following colors: red, orange, yellow, green, light blue, dark blue, and white. Then, we conducted a *post hoc* comparison to check which groups differed from each other. We used the Tukey HSD test for the *post hoc* comparison, and the results are also presented in Table 6.

**Table 6:** Comparison between five clusters’ color preference.

Color preference	Cluster 1 (*N* = 333)	Cluster 2 (*N* = 85)	Cluster 3 (*N* = 108)	Cluster 4 (*N* = 119)	Cluster 5 (*N* = 209)	*F*	*Post hoc* comparison
Red	0.16	−0.17	0.09	−0.24	−0.08	5.21^***^	1 > 2, 1 > 4, 1 > 5
Orange	0.18	−0.21	−0.19	−0.18	0.04	5.55^***^	1 > 2, 1 > 3, 1 > 4
Yellow	0.12	−0.21	−0.08	−0.09	0.03	2.53^*^	
Green	−0.07	−0.22	0.11	0.06	0.13	2.76^*^	2 < 5
Light blue	−0.18	0.11	0.09	0.28	0.07	6.05^***^	1 < 4, 1 < 5
Dark blue	−0.13	0.17	0.11	0.23	−0.04	4.14^**^	1 < 4
Purple	−0.09	0.04	0.15	−0.04	0.07	1.68	-
White	−0.20	0.15	0.14	0.16	0.04	5.32^***^	1 < 2, 1 < 3, 1 < 4, 1 < 5
Gray	−0.12	0.09	−0.10	0.04	0.07	1.80	
Black	−0.07	0.24	0.00	−0.05	−0.04	1.73	

^*^p < 0.05, ^**^p < 0.01, and ^***^p < 0.001.

## Discussion

Using quantitative data and statistical analysis, this study examined the relationship between personality and color preference. The main results confirmed that people have certain tendencies for the color connotations and associations. For instance, when participants heard the words “extraverted” or “enthusiastic,” they chose red most frequently, followed by warm colors such as orange and yellow. This result aligns with the contention that red represents energy, passion, strength, vigor, and physical activity (Verner-Bonds, 2000; Park and Song, 2014; Jue, 2017).

Red and yellow are long-wavelength colors, and green and blue are short-wavelength colors. Our analysis showed associations between long-wavelength warm colors and the two words representing extraversion and between short-wavelength or achromatic colors and the two words opposite to extraversion. These results imply that a more or less consistent correlation between color impressions and personality types.

While sympathy and warmth, which constitute agreeableness, were associated with yellow, the anti-agreeable qualities, critical and quarrelsome, were associated with intense colors that contrast with yellow. In particular, participants ranked red and black as the colors first- and second-most strongly associated with quarrelsomeness. Red-black is an especially sinister color combination known as rouge noir (Jue, 2017); it looks powerful and intense, represents antagonism, and evokes quarrelsomeness.

Indeed, red makes a strong impression. For that reason, participants selected it as the color that most strongly connotes anti-conscientiousness and emotional volatility. Such intensity, vitality, and attributes reminiscent of blood also manifested in the relationship between age and color preference. For instance, 33.3% of elderly females chose red as their most preferred color (Suh, 2011).

Meanwhile, participants chose green (red’s complementary color) as the color most strongly associated with conscientiousness and emotional stability. Green is usually found in nature and its connotations include fertility, growth, peace, and safety. A previous study identified “pure (28.6%)” and “natural (21.4%)” as adjectives that are frequently used to describe green (Park and Song, 2014). This offers a plausible explanation for the associations between green and dependable, self-disciplined, calm, emotionally stable, and openness to new experiences found in this study.

Blue was not the first color associated with personality adjectives, but it was chosen as the color second- or third-most strongly associated with reserved, critical, dependable, self-disciplined, calm, and emotionally stable. Sharpe (1974) pointed out that warm colors are exciting and stimulating, while cold colors are stable. Also, people who direct attention inward rather than outward are attracted to short-wavelength blue with the property of contraction. Likewise, we found that short-wavelength blue is associated with emotion-suppressing characteristics, while the long-wavelength red, located on the opposite spectrum, is associated with emotion-expressing characteristics (Holtzschue, 2011). Furthermore, through regression and cluster analyses, blue was found to be a valid predictive variable that distinguishes different personalities.

Many participants identified black as a color associated with the words conventional and uncreative. Black absorbs all light and does not reflect it, leaving a heavy and dark impression. One previous study found that in the context of object and clothing design, black gives a modern, noble, and refined impression (Choi, 2012; Park and Song, 2014), but when we presented it separately from objects, we found that it left a negative impression. Participants also selected gray and white as colors associated with the words conventional and uncreative. Gray is the color of ashes—the opposite of vitality. Nevertheless, we found that, like all colors, gray carried a mix of positive and negative associations, ranging from tranquility, wisdom, and intellect to disorganized and critical.

Through the correlation analysis and the regression analysis, we were able to examine the relationship between personality type and color preference. The results of both analyses were similar, except for a few colors. For instance, preferred colors that significantly predicted *conscientiousness* included light blue only, and excluded red and dark blue, which were found to be significant in the correlation analysis results. The difference between the correlation analysis results and the regression analysis results might be due to the standardization of scores; the latter used the standardized scores while the former used the original scores.

The regression analysis results showed that red and light blue preferences significantly predicted emotional stability. That is, as the strength of the red preference increased, conscientiousness decreased, and as the strength of the blue preference increased, the conscientiousness increased. This aligns with our personality–color association results and echoes findings of Cho and Oh (2016) regarding the relationship between psychological state and color preference. That is, the high-anxiety group chose “warm colors” such as red, orange, and yellow as their preferred colors, while the low-anxiety group preferred “cold colors” such as blue. Both the high- and low-anxiety groups reported that their color preferences and aversions stemmed from the feelings evoked by the colors.

We tested how color preferences change according to personality types in the following two ways. First, we divided participants into two groups based on the strength or weakness of their personality types, and then compared the groups’ color preferences. Second, we also divided the entire participants into five clusters through cluster analysis and compared their color preferences. There were significant differences in color preference according to personality clusters. In general, we found that those with stronger personalities tended to prefer the colors associated with their personalities. For example, those with strongly agreeable personalities preferred yellow, which is associated with that characteristic. A previous study examining the relationship between MBTI personality and color preference found that the preference for yellow was lower among thinking-type people than among emotional-type people (Cha and Jung, 2018). Since that agreeableness is a characteristic of emotional-type people, our results align with these past findings.

People with strong “open to new experiences” personalities had a more robust preference for green than those without this attribute. Our investigation of word-color associations showed the strongest association ratios of green and purple for the corresponding personality, and these results were also obtained in the regression analysis performed after standardizing the scores. Notably, the results show the relationship between a specific personality and its associated color, as do our findings regarding the relationships between blue and both conscientiousness and emotional stability. High conscientiousness participants preferred blue to a greater extent than low conscientiousness participants. Likewise, emotionally stable participants preferred light blue to a greater extent than unstable participants. Previously, participants chose blue as the color most closely associated with conscientiousness and emotional stability. In particular, they indicated that light blue symbolizes dependability and emotional stability, while dark blue symbolizes dependability, self-discipline, and calm. Similarly, in a previous study of color preference and anxiety levels, the low anxiety group preferred cold colors such as blue, while the high anxiety group preferred warm colors (Cho and Oh, 2016). Meanwhile, we found that emotionally stable participants disliked red the most. In a broad sense, this finding is consistent with results linking red and emotional volatility.

Park and Song (2014) explored why people prefer specific colors, and 76.3% of their participants answered that they liked the feeling they received from their favorite color. Obtained from college students in their 20s, these results echo those of a study focused on color experts (Kim and Park, 2022). For example, participants associated the word “modern” with the whole range of achromatic colors as well as blue; meanwhile, they associated “natural” with green and yellow. In this study, we investigated the colors 876 people between the ages of 20 and 60 years associated with personality words, and our results align with the findings of previous associative color studies.

Unexpectedly, we also found that extroverts prefer green over red. This contrasts with the tendency of other personality types to like the colors associated with their personalities. It also runs counter to the findings of previous studies showing that extraverts have high tolerance for light (Ludvigh and Happ, 1974), like high chroma colors (Pazda and Thorstenson, 2018), and generally prefer high-intensity stimuli. Indeed, although people generally associate extraversion with red, we found that extraverts prefer green—red’s complementary color of red. Do these results reflect the cultural characteristics of the East, which tend to be unfavorable for extroverts? For example, have Korean society’s efforts to teach extroverts temperance and emotional serenity affected them individually? Do extroverts need a color that adds stability and vitality to their passionate and extroverted sides? Or does red have any properties that make it more unique than other colors? For instance, people love red, but they might sometimes get tired of its intensity. Our findings provide only limited insight into extroverts’ color preferences, and we cannot exclude the possibility of cultural influence; future studies should endeavor to consider red’s multiple meanings and complex symbolism together.

This study’s limitations and suggestions for future studies are as follows. First, significant correlations were found between personality and color preference, but the correlation coefficients were fairly small, and thus the correlations were not very strong, only showing rough trends. Second, we measured the participants’ traits though a simple TIPI tool. Although TIPI has been reported to discriminate the big five personality traits well, it may be coarse compared to the detailed questionnaire. Third, because this study investigated the relationship between personality and color preference among Korean adults, the generalizability of its findings is limited. The fact that color preferences and senses of color often differ across ages, genders, and cultures highlights the need for explorations of the relationship between color preference and personality with people from diverse countries and regions. Fourth, we did not ask about the participants’ sexual orientation. Therefore, we may have omitted variables that could have influenced the analysis of color preference results.

## Data availability statement

The raw data supporting the conclusions of this article will be made available by the authors, without undue reservation.

## Ethics statement

This study was conducted at Hanyang Cyber University, Seoul, Republic of Korea, between August 2022 and September 2022. This study was approved by Hanyang Cyber University’s Institutional Review Board (Reference HYCU-IRB-2022-005). The patients/participants provided their written informed consent to participate in this study.

## Author contributions

JH: data curation, investigation, and project administration. JJ and JH: formal analysis. JJ: methodology, visualization, and writing—original draft. All authors contributed to the article and approved the submitted version.

## Funding

This work was supported by the National Research Foundation of Korea (NRF) grant funded by the Korea government (MSIT) (No. 2022R1/1A4053429).

## Conflict of interest

The authors declare that the research was conducted in the absence of any commercial or financial relationships that could be construed as a potential conflict of interest.

## Publisher’s note

All claims expressed in this article are solely those of the authors and do not necessarily represent those of their affiliated organizations, or those of the publisher, the editors and the reviewers. Any product that may be evaluated in this article, or claim that may be made by its manufacturer, is not guaranteed or endorsed by the publisher.
